# Distinct Effects of Familial Parkinson’s Disease-Associated Mutations on α-Synuclein Phase Separation and Amyloid Aggregation

**DOI:** 10.3390/biom13050726

**Published:** 2023-04-23

**Authors:** Bingkuan Xu, Fengshuo Fan, Yunpeng Liu, Yinghui Liu, Lin Zhou, Haijia Yu

**Affiliations:** 1 Jiangsu Key Laboratory for Molecular and Medical Biotechnology, College of Life Sciences, Nanjing Normal University, Nanjing 210023, China; 2 School of Chemistry and Bioengineering, Nanjing Normal University Taizhou College, Taizhou 225300, China

**Keywords:** α-synuclein, mutation, phase transition, amyloid aggregation, liquid–liquid phase separation (LLPS), Parkinson’s disease (PD)

## Abstract

The Lewy bodies and Lewy neurites are key pathological hallmarks of Parkinson’s disease (PD). Single-point mutations associated with familial PD cause α-synuclein (α-Syn) aggregation, leading to the formation of Lewy bodies and Lewy neurites. Recent studies suggest α-Syn nucleates through liquid–liquid phase separation (LLPS) to form amyloid aggregates in a condensate pathway. How PD-associated mutations affect α-Syn LLPS and its correlation with amyloid aggregation remains incompletely understood. Here, we examined the effects of five mutations identified in PD, A30P, E46K, H50Q, A53T, and A53E, on the phase separation of α-Syn. All other α-Syn mutants behave LLPS similarly to wild-type (WT) α-Syn, except that the E46K mutation substantially promotes the formation of α-Syn condensates. The mutant α-Syn droplets fuse to WT α-Syn droplets and recruit α-Syn monomers into their droplets. Our studies showed that α-Syn A30P, E46K, H50Q, and A53T mutations accelerated the formation of amyloid aggregates in the condensates. In contrast, the α-Syn A53E mutant retarded the aggregation during the liquid-to-solid phase transition. Finally, we observed that WT and mutant α-Syn formed condensates in the cells, whereas the E46K mutation apparently promoted the formation of condensates. These findings reveal that familial PD-associated mutations have divergent effects on α-Syn LLPS and amyloid aggregation in the phase-separated condensates, providing new insights into the pathogenesis of PD-associated α-Syn mutations.

## 1. Introduction

**Figure 1:**
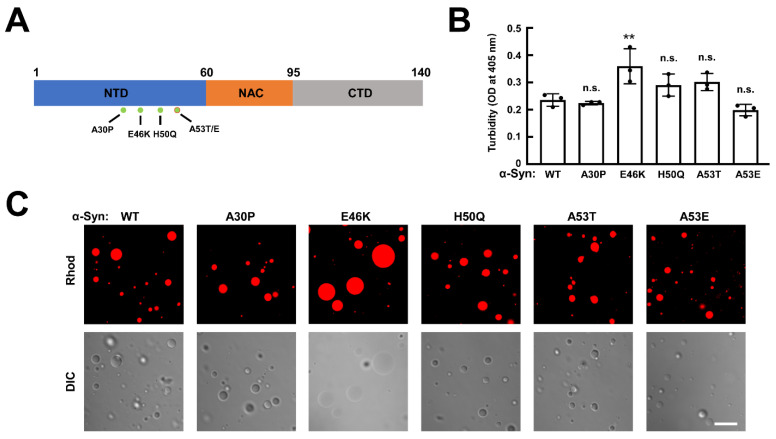
WT and familial PD-associated mutant α-Syn undergo LLPS in vitro. (**A**) Illustration showing the familial PD-associated mutations. The NTD, NAC, and CTD of α-Syn are indicated in different colors. (**B**) Turbidity assays showing the formation of phase-separated condensates by WT and mutant α-Syn. Turbidity was evaluated by monitoring the absorbance at 405 nm. Data are presented as mean ± SD (n = 3 independent replicates). *p* values were calculated using one-way ANOVA with Tukey’s multiple comparisons test. n.s., *p* > 0.05. **, *p* < 0.01. (**C**) Fluorescence and differential interference contrast (DIC) images showing the morphologies of Rhod-labeled WT and mutant α-Syn droplets. The α-Syn concentration is 200 μM. Scale bar, 5 μm. All the experiments were carried out in the presence of 20% PEG-10000.

The deposition of Lewy bodies and Lewy neurites is one of the key pathological hallmarks of Parkinson’s disease (PD) [1]. The amyloid aggregates of misfolded α-Syn are the main components of Lewy bodies and Lewy neurites, which links α-Syn lesions to PD [2]. Both soluble oligomers and insoluble fibrils are reported to contribute to α-Syn neurotoxicity [3,4,5]. α-Syn is a small cytoplasmic soluble protein composed of 140 amino acid residues, dividing into three domains: N-terminal domain (1–60 aa), non-amyloid-β component (NAC) domain (61–95 aa), and C-terminal domain (96–140 aa) (Figure 1A) [6,7]. α-Syn is primarily located at the presynaptic terminal [8], involved in vesicle recycling and synaptic remodeling. However, the exact physiological function of α-Syn is still largely unknown [9].

Multiple single-point mutations of α-Syn identified in familial PD, including A30P, E46K, H50Q, A53T, and A53E, were widely reported to lead to different early-onset pathology symptoms [10,11,12]. The molecular mechanisms of these mutations causing PD remains to be determined. One hypothesis is that amino acid replacements may alter the dynamics of α-Syn self-assembly into oligomers and fibrils. The conversion of α-Syn monomers to amyloid fibrils follows the nucleation-dependent polymerization pathway, including the lag stage, extension stage, and platform stage. A53T, the first discovered familial PD-associated mutation, dramatically accelerates α-Syn aggregation in vitro, demonstrated by a variety of biochemical methods [13,14]. It has been reported that A53T mutation induces PD-like pathology in cells and animal models [15,16]. Another identified familial PD-associated mutation that accelerates α-Syn aggregation is E46K, which disrupts the salt bridge and rearranges the fibrous structure to accelerate the aggregation [17,18]. H50Q, a mutation discovered in PD patients with a history of familial parkinsonism and dementia, promotes amyloid aggregation by reducing the solubility of monomers and the lag time of fibril formation in vitro [19,20,21]. Further studies suggest that the H50Q mutation stimulates SH-SY5Y cells to secrete α-Syn and shows higher toxicity to cultured hippocampal neurons than wild-type (WT) α-Syn [22]. A recent study reveals that the H50Q fibril has a different structure from WT α-Syn, leading to faster aggregation and stronger cytotoxicity [23]. The effect of A30P mutation on α-Syn aggregation is controversial. While some studies show that A30P accelerates the oligomerization of α-Syn but decelerates fibrillation, other reports suggest that A30P decreases the oligomerization [13,24,25,26,27]. Interestingly, the A30P mutant is more neurotoxic than WT α-Syn [28,29,30,31]. A53E is a recently identified missense mutation of α-Syn associated with PD [32,33]. Compared with WT α-Syn, the A53E mutant reduces the formation of amyloid aggregates in vitro but enhances the toxicity through mitochondrial stress [34,35]. Another study reveals that α-Syn A53E mutation increases proteasome activity to alter normal protein homeostasis [36]. The cell-based studies suggest that α-Syn A53E mutation enhances cell death in response to environmental toxins due to a larger population of oligomers [37].

Many intracellular membrane-less organelles, such as nucleoli, Promyelocytic leukemia nuclear bodies, and P bodies, are formed by liquid–liquid phase separation (LLPS) [38,39]. The phase-separated condensates are usually spherical droplets with liquid properties and high internal fluidity, enabling the exchange of molecules between the concentrated and diluted phases. Interestingly, the aggregation of many amyloid-prone proteins is initiated by LLPS, such as tau, RNA-binding protein fused in sarcoma (FUS), and α-Syn [40,41,42]. Recent studies showed α-Syn undergoes LLPS in vitro and further forms amyloid aggregates through nucleation in the condensates [42,43]. The formation of α-Syn liquid-like condensates was observed in a C. elegans model of PD [44]. During LLPS, α-Syn takes an “elongated” conformation with a high local concentration, facilitating intermolecular aggregation [45]. We showed that the C-terminal domain has a strong effect on α-Syn LLPS through the regulation of electrostatic interactions [46]. α-Syn-interacting factors, including lipids, dopamine, and metal ions, could modulate the phase separation of α-Syn [42,47]. Most of the familial PD-associated mutations take effect on the amyloid aggregation of α-Syn. How these mutations regulate α-Syn LLPS and the subsequent amyloid aggregation in condensates remains to be determined.

In this work, we examined the effects of five previously identified mutations in familial PD on the LLPS of α-Syn. While WT and all the mutated α-Syn proteins undergo LLPS in vitro, the E46K mutation dramatically enhances the formation of condensates. The high fluidity of the WT and mutant α-Syn condensates confirms that the initially formed condensates are liquid-like. Further studies have shown that the droplets of α-Syn mutants fuse with WT α-Syn droplets and recruit WT monomers. We observed that α-Syn underwent a liquid-to-solid phase transition in the condensates, indicating the formation of amyloid aggregation. The A30P, E46K, H50Q, and A53T mutations accelerated the phase transition and the amyloid aggregation. In contrast, the A53E mutation retarded this process, showing a distinct effect. Finally, we observed the condensates in the cells expressing WT α-Syn or the mutants. Consistent with the in vitro study, the E46K mutation increases the formation of condensates in the cells, suggesting the mutation of E46K promotes amyloid aggregation by facilitating the LLPS of α-Syn. Together, our studies suggest that the mutations from familial PD play distinct roles in α-Syn phase separation and the amyloid aggregation in the phase-separated condensates, which may be implicated in mutagenesis-caused PD.

## 2. Materials and Methods

### 2.1. Protein Expression and Purification

Recombinant untagged α-Syn was expressed in *E. coli* and purified by nickel affinity chromatography as previously described [48,49,50,51]. The cDNA encoding the human α-Syn gene was cloned into a pET28a-based SUMO vector [48]. The recombinant plasmid was transformed into a BL21 (DE3) competent cell. The His_6_-SUMO-α-Syn fusion protein was expressed by induction using 1 mM isopropyl β-D-galactopyranoside (IPTG) when the OD_600_ of culture reached 0.6–0.8. After another 2 h of induction at 37 °C, the bacteria were harvested by centrifugation at 8000× *g* for 10 min at 4 °C. The pellets were resuspended in 40 mL of lysis buffer (25 mM HEPES [pH 7.4], 150 mM KCl, and 20 mM imidazole) containing 1 mM PMSF and protease inhibitor cocktail (Roche, one tablet per 100 mL buffer). The bacteria were lysed using homogenization. The lysates were then centrifuged at 18,000× *g* for 30 min at 4 °C, and the His_6_-SUMO-α-Syn fusion protein in the supernatant was purified by nickel affinity chromatography. The extra His_6_-SUMO tags were removed by cleavage of SUMO protease. The purified untagged protein was subsequently dialyzed overnight against a protein storage buffer (25 mM Tris-HCl [pH 7.4], 50 mM NaCl). The monomeric α-Syn protein was characterized by gel filtration chromatography and Circular Dichroism (*CD*) spectroscopy.

For EGFP-α-Syn protein preparation, the cDNA encoding EGFP and α-Syn were incorporated into a pET28a-based SUMO vector. The EGFP-α-Syn fusion protein was expressed and purified following the same procedure as untagged α-Syn [48]. α-Syn mutants were generated by site-directed mutagenesis and purified similarly to WT protein.

### 2.2. Protein Labeling

α-Syn was fluorescence-labeled by a rhodamine labeling kit following the manufacturer’s instructions (ThermoFisher Scientific, Waltham, MA, USA). Briefly, 1 mL α-Syn protein at a concentration of 2 mg/mL was dialyzed against the labeling buffer (50 mM sodium borate, pH 8.5) and mixed with 10-fold molar excess of rhodamine dye. After incubation at room temperature for 1 h, the excess dye was removed by dialysis against the protein storage buffer at 4 °C for 24 h. The dialysis buffer was changed every 4 h [48,52].

### 2.3. Confocal Imaging and FRAP Assay

Unlabeled and fluorescence-labeled WT or mutant α-Syn were mixed at a molar ratio of 9:1 and 200 μM α-Syn mixture was incubated in the presence of 20% PEG-10000 (*w*/*v*) to form droplets. For image acquisition, each 5 μL sample was dropped on a glass slide, covered with a 14 mm coverslip and visualized with a 100 X oil immersion objective under a Nikon A1 microscope (Nikon Corporation, Japan).

For FRAP measurements, 5 μL of the fluorescence-labeled α-Syn condensates were dropped onto a glass slide. FRAP was performed using a Nikon A1 microscope with a 561 nm laser. The measurements involved one pre-bleaching frames, one flash of bleaching (100% of laser power), and twenty postbleaching frames. Data were normalized to the maximal prebleach and minimal postbleach fluorescence intensities.

### 2.4. Turbidity and ThT Fluorescence Assay

The turbidity was employed to evaluate the protein phase separation. Turbidity measurements were performed using a BioTek Synergy HT microplate reader. Then, 200 μM WT and mutant α-Syn immediately underwent LLPS to form condensates in the presence of 20% PEG-10000. The α-Syn WT and mutant samples were loaded into a transparent 96-well plate. The absorbance at 405 nm was measured at 37 °C immediately [48,52]. For the kinetics assay, the samples were incubated at 37 °C without shaking, and OD measurements were carried out at each time point. Full accounting of statistical significance was included for each datum based on at least three independent experiments.

The amyloid aggregation of α-Syn was indicated by ThT fluorescence. The ThT fluorescence assay was performed as previously described [48,52]. Then, 200 μM WT or mutant α-Syn droplets were incubated at 37 °C without shaking. The samples were diluted 40-fold, and the final concentration of ThT was 40 μM. Fluorescence was measured every 6 h on a BioTek Synergy HT microplate reader with an excitation wavelength of 440 nm and an emission wavelength of 485 nm.

### 2.5. Droplet–Droplet Fusion and α-Syn Monomer Recruitment Assay

For the measurement of droplet–droplet fusion between WT α-Syn droplets and mutant α-Syn droplets, EGFP-labeled WT α-Syn liquid droplets and rhodamine-labeled mutant α-Syn liquid droplets were prepared in the presence of 20% PEG-10000, respectively. The WT α-Syn droplets and mutant α-Syn droplets were gently mixed at the molar ratio of 1:1 and observed with a 100 X oil immersion objective under a Nikon A1 microscope at 488 nm and 561 nm.

We then developed the assays to measure the recruitment of WT α-Syn monomers to mutant α-Syn liquid droplets. Then, 200 μM rhodamine-labeled α-Syn mutants underwent LLPS to form liquid droplets in the presence of 20% PEG-10000. Subsequently, 10 μM EGFP-labeled WT α-Syn, which could not form liquid droplets at the concentration, was added to the liquid droplets formed by α-Syn mutants. The samples were gently mixed and observed with a 100 X oil immersion objective under a Nikon A1 microscope at 488 nm and 561 nm.

### 2.6. ThS Staining Assay

ThS solution was prepared at a concentration of 0.0625% in protein storage buffer. For each sample, 10 μL of α-Syn WT and mutant condensates were incubated with 1 μL ThS solution at 37 °C for 0 h, 24 h, and 48 h. ThS fluorescence was visualized with a 100 X oil immersion objective under a Nikon A1 microscope at 488 nm [48]. In order to exclude the background fluorescence and highlight the ThS fluorescence signal in the α-Syn condensates, all the ThS images at 0 h, 24 h, and 48 h were obtained under the same low laser power (10%).

### 2.7. CD Spectroscopy

Near UV CD spectra were measured using a Chirascan spectropolarimeter at 25 °C with a 1 mm quartz cell. The readings were made at 1 nm intervals, and each data point represented the average of five scans at a speed of 50 nm/min over the wavelength range of 200–260 nm. The concentration of α-Syn protein is 15 μM. The data were converted into mean residue weighted molar ellipticity using the following equation: [*θ*]_MRW_ = (100 × *θ*)/*Cnl*, where *C* is the protein concentration (mM), *θ* is the measured ellipticity (milli-degree), *n* is the number of residues, and *l* is the path length (cm).

### 2.8. Cell Culture and Transfection

HeLa cells were maintained in Dulbecco’s Modified Eagle Medium (DMEM) supplemented with 10% fetal bovine serum, 100 units/mL of penicillin, and 100 μg/mL of streptomycin in a water-saturated atmosphere of 5% CO_2_ at 37 °C [53]. Before transfection, HeLa cells were seeded onto a 15-mm confocal-dedicated glass-bottom cell culture dish (NEST Biotechnology Co.LTD, China) and allowed to grow up to 60–80% confluence. The cells were transfected with same amounts of indicated pmCherry C1 empty vector, pmCherry C1-α-Syn WT, or mutant constructs using Lipofectamine^®^ 2000 Reagent. After transfection, the cells were continuously cultured for another 36 h. The α-Syn condensates were visualized at 561nm with a 100 X oil immersion objective under a Nikon A1 microscope. The exposure time was controlled at a similar level.

### 2.9. Statistical Analysis

All data were presented as the mean ± SD and were analyzed using GraphPad Prism 8.0.2 software for Windows. Statistical significance was calculated using one-way ANOVA, and *p*-value < 0.05 was considered statistically significant.

## 3. Results

### 3.1. α-Syn and Its Familial PD-Associated Mutants Behave LLPS In Vitro

We expressed and purified recombinant WT α-Syn and familial PD-associated α-Syn mutants, A30P, H50Q, E46K, A53T, and A53E, to examine their behaviors in phase separation (Figure 1A). Using the turbidity assay, which measures the phase-separated condensates, we observed that all WT and mutant α-Syn proteins underwent LLPS in the presence of PEG, a common crowding agent used to initiate α-Syn phase separation [48,52]. The E46K mutation increased the turbidity significantly, while all four other mutations had little change (Figure 1B).

We then labeled the WT and mutant proteins with NHS-rhodamine [48]. Rhodamine-labeled and unlabeled α-Syn were mixed at a molar ratio of 1:9 to form droplets [48,52]. Using confocal microscopy, we directly observed that all the α-Syn proteins formed spherical condensates in the presence of 20% PEG-10000 (Figure 1C and Figure S1). The size of the condensates formed by the α-Syn E46K mutant was significantly larger than WT α-Syn condensates (Figure 1C and Figure S1). However, no noticeable size difference was observed for the other four α-Syn mutants, consistent with the turbidity assay (Figure 1C and Figure S1). We then analyzed the numbers and total area proportion of the α-Syn WT and mutant droplets in the fluorescence images. The E46K mutation has little effect on the number of α-Syn droplets. The total area of E46K droplets was largely higher than that of WT and the other mutations, supporting that E46K significantly facilitated the LLPS of α-Syn (Figure S2). Together, these studies suggest that E46K mutation promotes the LLPS of α-Syn, while the single mutation of A30P, H50Q, A53T, or A53E has little effect on the initial formation of α-Syn condensates [42,52].

### 3.2. The Familial PD-Associated α-Syn Mutations Do Not Affect the Fluidity of α-Syn Condensates

**Figure 2:**
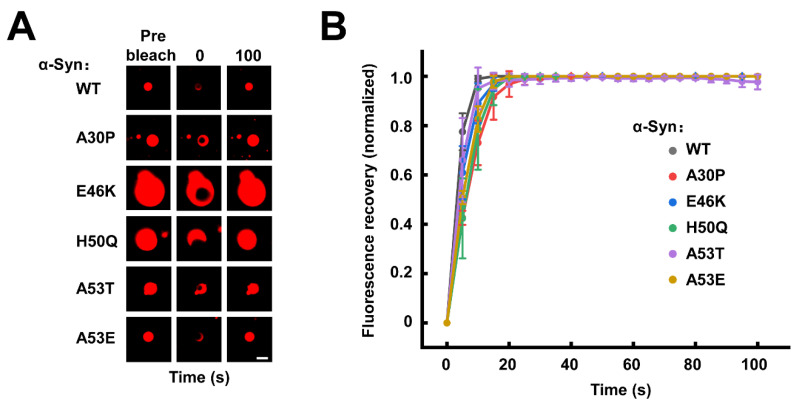
The familial PD-associated mutations do not affect the fluidity of α-Syn condensates. (**A**) Representative FRAP images of WT and mutant α-Syn condensates. The fluorescence images of prebleached, bleached (0 s), and bleached after 100 s recovery are shown. Scale bar, 2 μm. (**B**) The normalized FRAP curves of α-Syn condensates shown in A. Data are presented as mean ± SD (n = 3 independent replicates) and normalized to the maximal prebleach and minimal postbleach fluorescence intensities. The concentration of α-Syn is 200 μM. All the experiments were carried out in the presence of 20% PEG-10000.

The liquid droplets formed by LLPS are characterized by fluidity, which allows molecule movement inside the condensates and exchange between the concentrated and diluted phases [54,55,56]. Then, we employed fluorescence recovery after photobleaching (FRAP) assays to study the dynamics of α-Syn WT and mutants inside the condensates. After the laser bleaching of the WT α-Syn condensates, the fluorescence intensity of the bleached area was rapidly and completely recovered to their pre-bleaching state within 100 s, confirming the rapid fluidity of α-Syn inside the droplets (Figure 2A,B) [48,52,57]. A similar FRAP was observed in all the droplets formed by α-Syn mutants (Figure 2 and Figure S3), suggesting familial PD-associated α-Syn mutations do not hinder the mobility of α-Syn molecules in the initially formed droplets.

### 3.3. The Condensates of α-Syn Mutants Fuse with WT α-Syn Droplets and Recruit α-Syn Monomers

**Figure 3:**
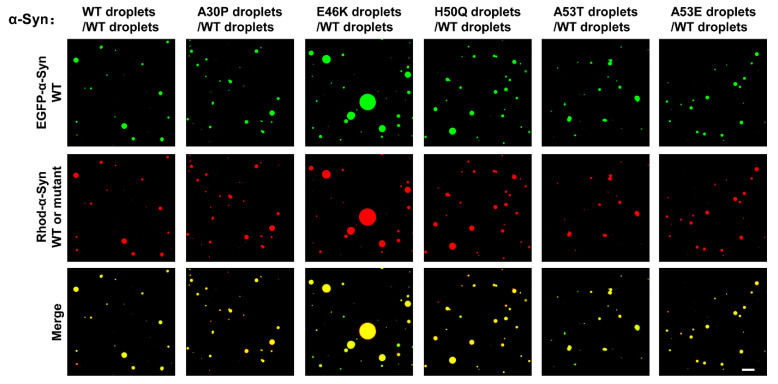
The mutant and WT α -Syn droplets can fuse. Representative images showing the fusion of two color-labeled droplets. The EGFP-labeled WT α-Syn droplets and Rhod-labeled WT and mutant α-Syn droplets were gently mixed and visualized through a confocal microscope. Scale bar, 5 μm. The experiments were performed in the presence of 20% PEG-10000.

The heterozygous point mutations of α-Syn are one of the crucial factors leading to familial PD. We then investigated the interplay between the mutant and WT α-Syn proteins during phase separation. The EGFP-α-Syn WT fusion protein was expressed and used to prepare the EGFP-labeled α-Syn droplets [48]. When we mixed the EGFP-labeled WT α-Syn droplets with Rhod-labeled WT or mutant α-Syn droplets, respectively, the α-Syn droplet–droplet fusion was universally observed. These data suggest that all the condensates formed by familial PD-associated α-Syn mutants could crossly fuse with WT droplets (Figure 3) [48].

**Figure 4:**
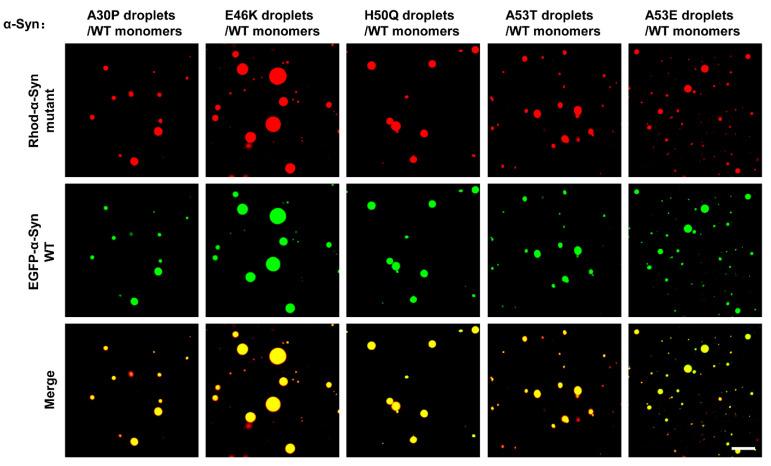
The familial PD-associated mutant α-Syn droplets recruit α-Syn monomers. Representative images showing the entry of EGFP-labeled α-Syn monomers into Rhod-labeled mutant α-Syn droplets. The total concentrations of α-Syn mutants are 200 μM, and the concentration of EGFP-labeled α-Syn monomers is 10 μM. Scale bar, 5 μm. The experiments were performed in the presence of 20% PEG-10000.

Next, we tested whether the liquid droplets formed by α-Syn mutants could act as a template to recruit WT α-Syn monomers, which were characterized by gel filtration chromatography (Figure S4A). The CD measurement showed a typical random coil structure, consistent with the reported α-Syn monomer structure (Figure S4B) [42]. When we added EGFP-labeled WT α-Syn monomers into the preformed Rhod-labeled mutant α-Syn droplets, the WT α-Syn monomers were integrated inside the droplets freely (Figure 4). Tau2N4R has been reported to interact with α-Syn and undergo LLPS [58,59]. We found that α-Syn droplets could fuse with Tau2N4R droplets and recruit free Tau2N4R into the droplets (Figure S5). Together, our data showed that the droplets formed by α-Syn mutants could not only fuse with WT α-Syn droplets but also recruit α-Syn monomers, demonstrating that an interplay occurred between WT and mutant α-Syn under phase separation.

### 3.4. Different Effects of Familial PD-Associated α-Syn Mutations on the Amyloid Aggregation in the Condensates

**Figure 5:**
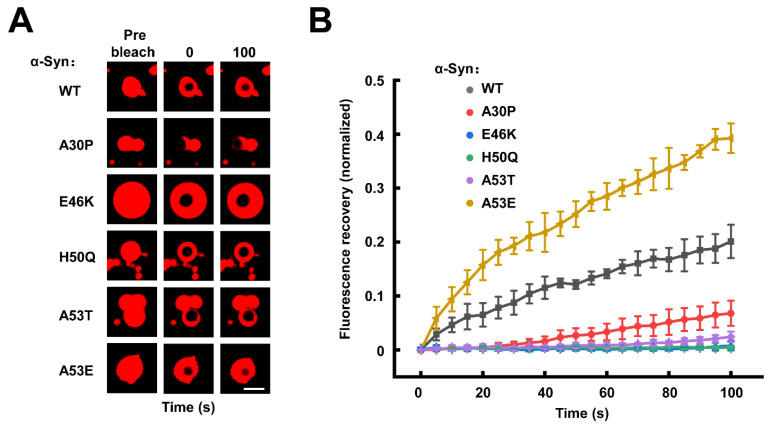
α-Syn undergoes a liquid-to-solid phase transition differently regulated by familial PD-associated mutations. (**A**) Representative FRAP images of WT and mutant α-Syn condensates after 48 h incubation. The fluorescence images of prebleached, bleached (0 s), and bleached after 100 s recovery are shown. Scale bar, 2 μm. (**B**) The normalized FRAP curves of α-Syn condensates shown in A. Data are presented as mean ± SD (n = 3 independent replicates) and normalized to the maximal prebleach and minimal postbleach fluorescence intensities. The concentration of α-Syn is 200 μM. All the experiments were carried out in the presence of 20% PEG-10000.

Recent studies suggest that phase separation facilitates α-Syn liquid-to-solid transition and accelerates amyloid aggregation inside the condensates [42,44]. We incubated the condensates formed by α-Syn WT or mutants for 48 h and examined their fluidity by the FRAP assay. The fluorescence intensity of condensates formed by α-Syn WT and mutants could not be recovered to the pre-bleaching, suggesting that the WT and mutant α-Syn were turning to a solid-like state after incubation (Figure 5A,B). However, their recovery rates were largely different. Compared with WT α-Syn condensates, the FRAP rate of the condensates formed by the A53E mutant was measurably higher. In contrast, the FRAP of A30P, A53T, E46K, and H50Q mutants was apparently lower (Figure 5B). These studies suggest all the familial PD-associated mutations except A53E accelerate the rates of liquid-to-solid transition, whereas A53E retards the phase transition in the phase-separated condensates.

**Figure 6:**
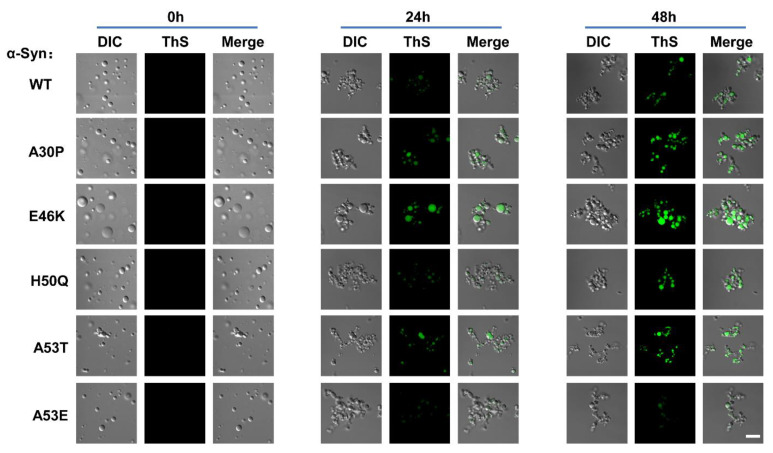
Distinct effects of familial PD-associated mutations on the formation of amyloid aggregates in the condensates. ThS staining images showing the amyloid structure inside the α-Syn condensates. WT or mutant α-Syn condensates were incubated with ThS for 0 h (**Left**), 24 h (**Middle**), and 48 h (**Right**), respectively. Scale bar, 5 μm. The concentration of α-Syn is 200 μM. The experiments were performed in the presence of 20% PEG-10000.

Next, we examined whether α-Syn forms amyloid aggregates during the phase transition using the Thioflavin S (ThS) staining assay, which is commonly used for amyloid staining in condensates [42,47,60]. No detectable ThS fluorescence signal was observed at the initial stage of phase separation, indicating that α-Syn did not form amyloid aggregates at the beginning of LLPS (Figure 6). After 24 h incubation, we observed the ThS fluorescence signal, which was substantially improved at 48 h, indicating the formation of amyloid structure over time in the condensates (Figure 6). Interestingly, the ThS fluorescence of A30P, E46K, H50Q, and A53T mutants in the condensates was stronger than WT α-Syn and the ThS fluorescence of A53E mutant was weaker, confirming the correlation between the liquid-to-solid phase transition and amyloid aggregation.

**Figure 7:**
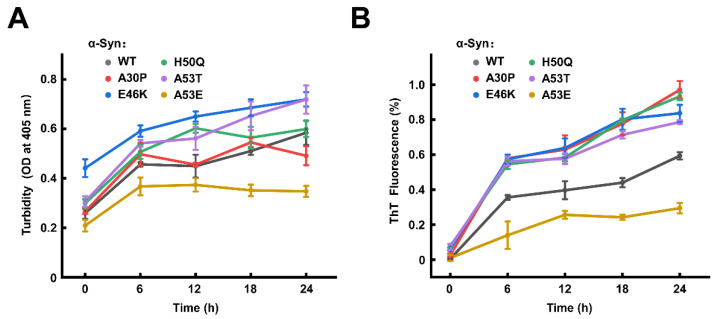
The familial PD-associated mutations have different effects on the amyloid aggregation of α-Syn in the condensation pathway. Kinetic curves of turbidity (**A**) and ThT fluorescence intensity (**B**) are shown. Data are presented as mean ± SD (n = 3 independent replicates). In the ThT assays, data are normalized to the maximal and minimal average fluorescence intensities. The concentrations of α-Syn are 200 μM. All the experiments were carried out at 37 °C without shaking in the presence of 20% PEG-10000.

We then measured the bulk solution turbidity and ThT fluorescence in the first 24 h to further compare the effects of mutations on α-syn LLPS and amyloid aggregation. The turbidity of α-Syn WT and five mutants increased continuously over time (Figure 7A). The ThT fluorescence intensity of α-Syn WT and mutants increased with the incubation time, suggesting the formation of the amyloid structure inside the condensates. Consistent with the ThS assay, A30P, E46K, H50Q, and A53T mutations accelerated the formation of α-Syn amyloid aggregates in the condensation pathway while the A53E mutant retarded the aggregation (Figure 7B). Taken together, our findings demonstrated that familial PD-associated mutations play distinct roles in forming amyloid aggregates, either promoting or retarding amyloid aggregation in the condensates, via the regulation of the liquid-to-solid phase transition.

### 3.5. The E46K Mutation Promotes the Formation of Condensates in the Cells

**Figure 8:**
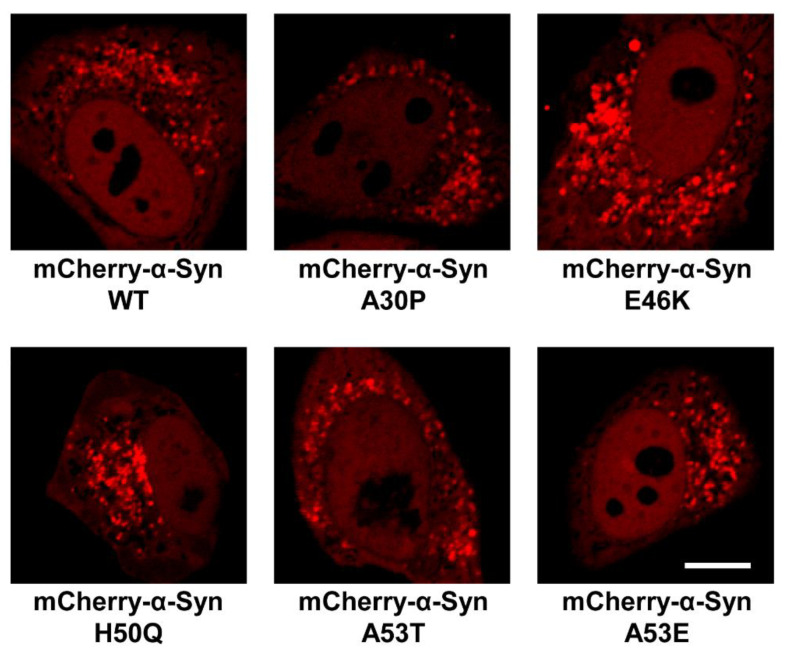
The E46K mutation promotes the formation of α-Syn condensates in cells. Representative confocal images of HeLa cells expressing WT and mutant mCherry-α-Syn, respectively. Scale bar, 5 μm.

While most studies examine α-Syn phase separation using the recombinant protein, fewer studies were performed in cells [42,47,57,61]. Next, we examined whether the WT and mutant α-Syn form condensates in the cultured cells. In a negative control experiment, we transfected the pmCherry C1 empty vector into Hela cells. The mCherry fluorescence was diffusely distributed throughout the whole cell with no condensates observed (Figure S6). Then, mCherry-tagged α-Syn WT and mutants were expressed in HeLa cells, respectively. We observed the condensates in the cells expressing WT α-Syn, consistent with previous reports [42]. Furthermore, the fluorescence-labeled condensates were also observed in HeLa cells expressing A30P, E46K, H50Q, A53T, or A53E mutants (Figure 8). Strikingly, the E46K mutation obviously promoted the formation of α-Syn condensates, supporting that it facilitates α-Syn LLPS dramatically.

## 4. Discussion

It has been established that the amyloid aggregation of α-Syn is closely related to the pathogenesis of PD [62,63,64]. The α-Syn oligomers and fibrils have a variety of cytotoxicity and neurotoxicity, which leads to the disease progression of PD [3,4,5,65]. The assemblies of α-Syn are considered promising drug targets for PD therapy. Hence, the amyloid aggregation of α-Syn have attracted much attention in the past few decades. Among the three domains, the central hydrophobic NAC domain primarily drives the intermolecular assembly, whereas the flanking domains play a further regulatory role. In the natural state, α-Syn shields the NAC domain and prevents aggregation through the long-range intramolecular interaction between the positively charged N-terminal and negatively charged C-terminal domains [66]. Multiple factors contributing to PD, such as heavy metal ions and abnormal phosphorylation, are able to change the kinetics of α-Syn aggregation by altering the charge balance to expose the hydrophobic NAC domain [67].

The familial PD-associated mutations of α-Syn are key factors leading to PD. Several familial PD-associated single-point mutations have been successively identified in recent decades, including A53T, A30P, E46K, H50Q, and A53E [62]. Multiple studies confirmed that the A53T, E46K, and H50Q mutations could accelerate the kinetics of α-Syn amyloid aggregation in vitro, although their mechanisms are not identical [13,17,20]. The mutation of A53E was reported to retard the amyloid aggregation of α-Syn, while the effect of A30P mutation remains incompletely understood [13,24,25,26,27,34]. Many lines of evidence suggest that α-Syn may take two pathways to form amyloid aggregates. One is the traditional deposition pathway, and the other is the recently discovered condensation pathway [42,44]. While the roles of familial PD-associated mutations in α-Syn amyloid aggregation have been widely studied in the deposition pathway, the effects of α-Syn mutations on amyloid aggregation under the condensation pathway remain to be determined.

This work comprehensively examined the effects of five mutations commonly discovered in familial PD on α-Syn LLPS. Our data suggested that the E46K mutation significantly promotes α-Syn LLPS, consistent with the previous study [42]. We observed that α-Syn undergoes a liquid-to-solid phase transition to form amyloid aggregation in the condensation pathway. Interestingly, the familial PD-associated mutations show divergent effects on this phase transition process. While all four other mutations, including E46K, promote the phase transition and amyloid aggregation, the A53E mutation apparently retards α-Syn aggregation in the condensation pathway.

Heterozygous α-Syn missense mutations exist in most patients with familial PD [11,32,68,69]. The fibrils formed by mutants could template WT α-syn monomers to form cross-seeding amyloid fibrils, inheriting the structure and pathological properties of mutants [18]. These studies suggest the interplays between WT and mutant α-Syn play critical roles in the progression of PD. We observed that the liquid droplets formed by α-Syn mutants could crossly fuse with WT α-Syn droplets, providing evidence for the interactions of familial PD-associated α-Syn mutants with WT α-Syn in the phase-separated condensates. The recruitment of WT α-Syn monomers into the mutant condensates further suggests that the formation of cross-seeding α-Syn aggregates could be facilitated by the phase separation.

In this cell-based study, we observed that the cells expressing the α-Syn E46K mutant generate more condensates than those expressing WT α-Syn or the other four mutants, consistent with the findings in LLPS assays using recombinant proteins. Why does E46K have such a substantial effect on α-Syn phase separation? Previous studies suggest E46K mutation disrupts the salt bridge between E46 and K80 [17]. We recently showed that α-Syn phase separation is highly regulated by electrostatic interactions [46]. Therefore, we postulate that the E46K mutation may promote α-Syn phase separation via the alternation of electrostatic interactions, facilitating the intermolecular associations. Since the roles of the LLPS in α-Syn aggregation in vivo and PD pathogenesis remain elusive, the relevance of our studies using the recombinant WT and mutant α-syn proteins with the pathogenesis of familial PD-associated α-syn mutations needs to be further determined. Furthermore, the effects of sporadic PD-related mutations on α-Syn LLPS and amyloid aggregation need to be studied. 

Taken together, these findings suggest α-Syn familial PD-associated mutations have divergent effects on α-Syn phase separation and subsequent amyloid aggregation in the condensation pathway. The mutants interact with WT α-Syn by the manners of droplet fusion or monomer recruitment, which puts forward a new insight into the crosstalk between WT and PD-associated mutant α-Syn under LLPS.

## 5. Conclusions

In this work, we investigated the effects of five familial PD-associated mutations, A30P, E46K, H50Q, A53T, and A53E, on the phase separation and phase transition of α-Syn. By using confocal imaging, turbidity, FRAP, and cell-based assays, we showed that the E46K mutation substantially promotes the α-Syn phase separation, while four other mutations have little effect on the initial formation of α-Syn condensates. α-Syn A30P, E46K, H50Q, and A53T mutations accelerate the amyloid aggregation in the phase-separated condensates. In contrast, the α-Syn A53E mutation retarded the aggregation during the liquid-to-solid phase transition. The droplet–droplet fusion assay and α-Syn monomer recruitment experiments suggested that the mutant α-Syn droplets could fuse to WT α-Syn droplets and recruit α-Syn monomers into their droplets, demonstrating that an interplay occurred between WT and mutant α-Syn under phase separation. Our findings reveal that familial PD-associated mutations have distinct effects on α-Syn LLPS and amyloid aggregation, providing new insights into the pathogenesis of PD-associated α-Syn mutations.

## Data Availability

The data are contained within the article.

## Supplementary Materials

The following supporting information can be downloaded at: https://www.mdpi.com/article/10.3390/biom13050726/s1, Figure S1: Additional fluorescence images of WT and mutant α-Syn droplets corresponding to Figure 1C; Figure S2: The quantified numbers (A) and total area proportion (B) of WT and mutant α-Syn droplets in the fluorescence images; Figure S3: The FRAP of α-Syn E46K droplets with small size; Figure S4: Characterization of α-Syn protein by gel filtration chromatography and CD spectroscopy; Figure S5: α-Syn droplets fuse with Tau2N4R droplets and recruit free Tau2N4R protein; Figure S6: Representative confocal image of HeLa cells transfected with pmcherry C1 empty vector.
