# Supplementary material for: Distinct Effects of Familial Parkinson’s Disease-Associated Mutations on α-Synuclein Phase Separation and Amyloid Aggregation
Source: Biomolecules. 2023 Apr 23;13(5):726. doi: 10.3390/biom13050726 (PMC10216457; doi:10.3390/biom13050726)
Supplement: Supplementary file 1 [file biomolecules-13-00726-s001.zip › biomolecules-2309251-supplementary.pdf]

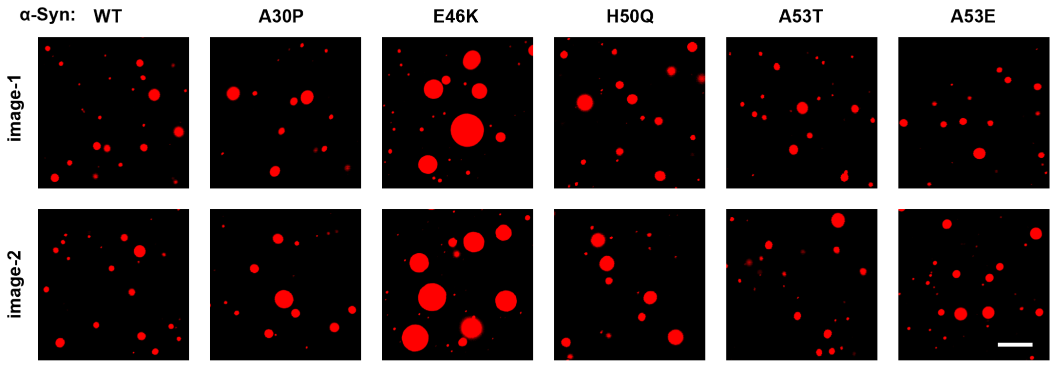

Figure S1. Additional fluorescence images of WT and mutant α-Syn droplets corresponding to Figure 1C. Scale bar, 5 μm.

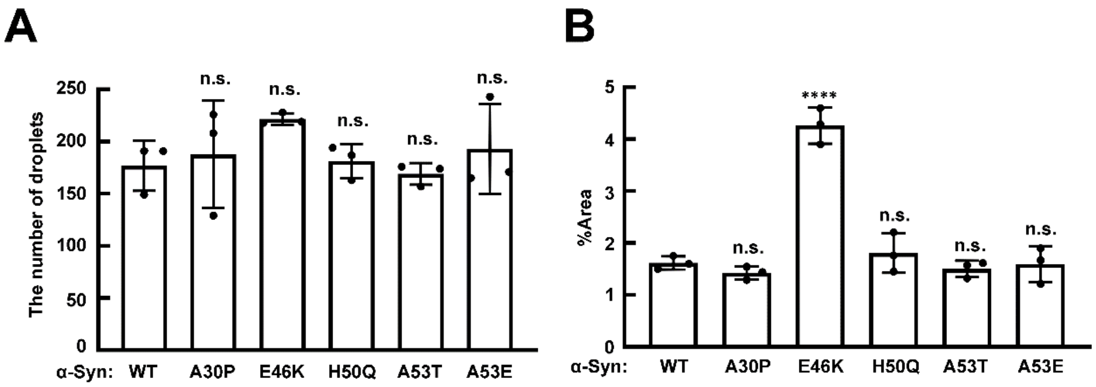

Figure S2. The quantified numbers (A) and total area proportion (B) of WT and mutant α-Syn droplets in the fluorescence images. The total concentrations of α-Syn WT and mutations are both 200 μM. Three independent confocal images were analyzed by ImageJ. Data are presented as mean ± SD (n = 3 independent replicates). P values were calculated using one-way ANOVA with Tukey's multiple comparisons test. n.s., $P > 0.05$. ****, $P < 0.0001$.

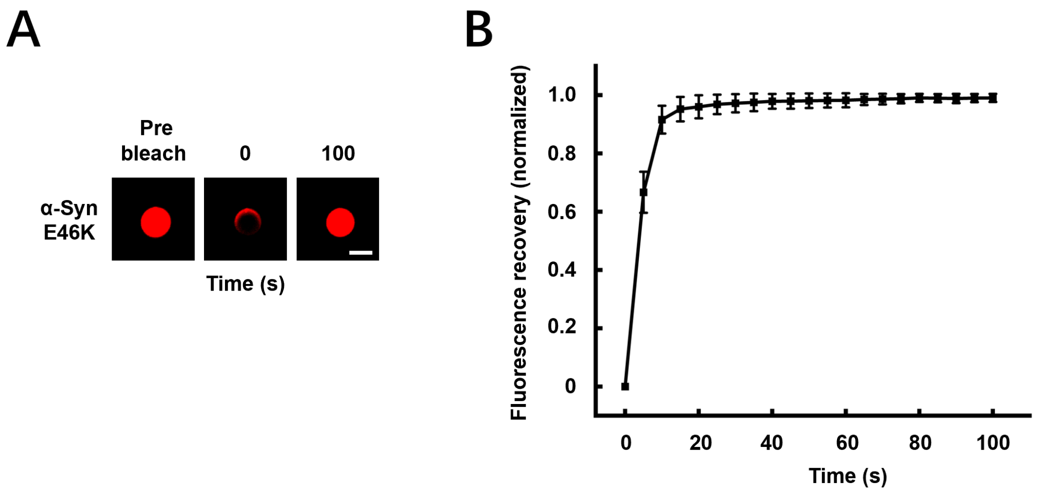

Figure S3. The FRAP of α-Syn E46K droplets with small size. (A) Representative FRAP images of α-Syn E46K droplets with small size. The fluorescence images of prebleached, bleached (0 s), and bleached after 100 s recovery are shown. Scale bar, 2 μm. (B) The normalized FRAP curves of α-Syn E46K droplets shown in A. Data are presented as mean ± SD (n = 3 independent replicates) and normalized to the maximal prebleach and minimal postbleach fluorescence intensities. The concentration of α-Syn E46K mutant is 200 μM and the experiments were carried out in the presence of 20% PEG-10000.

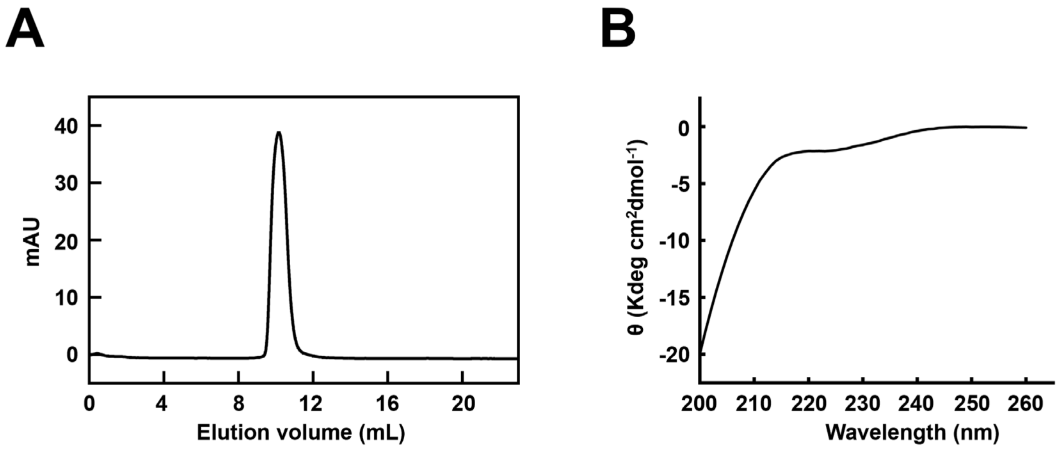

Figure S4. Characterization of α-Syn protein by gel filtration chromatography and CD spectroscopy. (A) The gel filtration chromatography of purified α-Syn protein showing a single elution peak using Superdex 75 Increase 10/300 GL. (B) CD measurement showing a random coil structure of α-Syn.

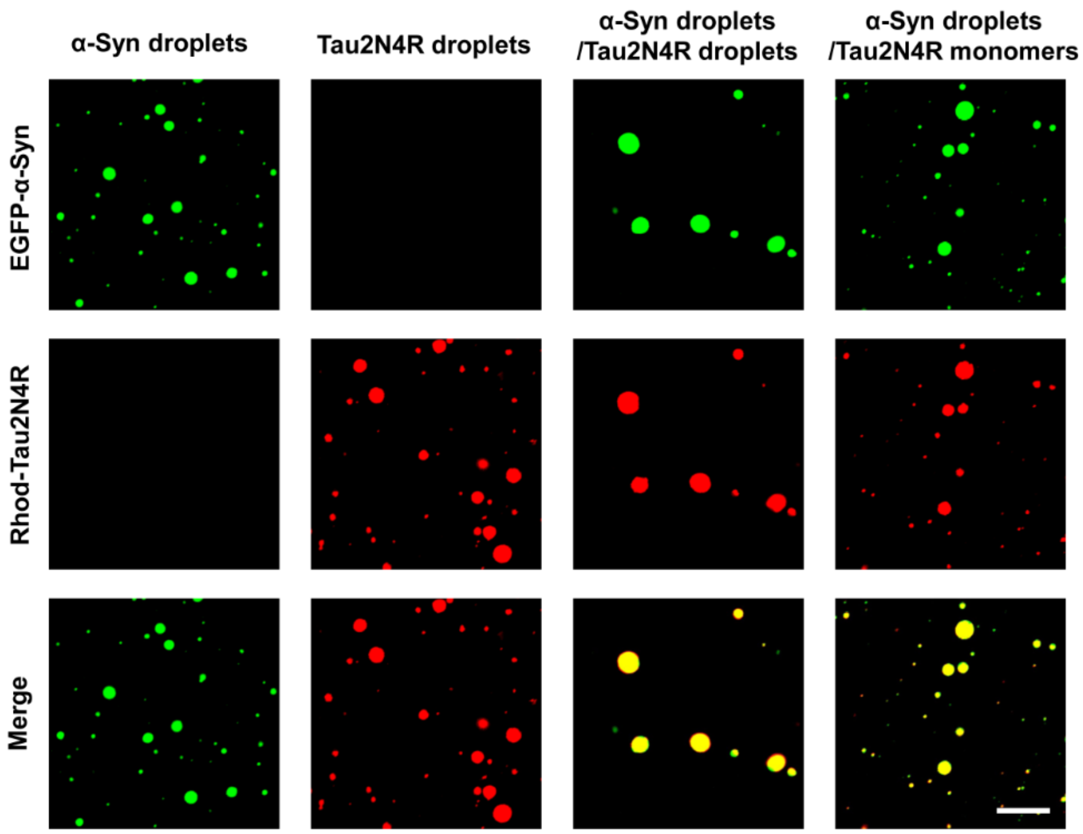

Figure S5. α-Syn droplets fuse with Tau2N4R droplets and recruit free Tau2N4R protein. Representative images showing the fusion between α-Syn droplets and Tau2N4R droplets and the recruitment of Tau2N4R monomers by α-Syn droplets. The molar ratio of EGFP-labeled α-Syn and Rhod-labeled Tau2N4R to unlabeled protein is 1:9, respectively. The total concentrations of α-Syn and Tau2N4R are 200 μM and 40 μM to form droplets in the presence of 20% PEG-10000, respectively. For recruitment experiment, 2 μM Rhod-labeled Tau2N4R monomers were added to preformed EGFP-labeled α-Syn droplets. Scale bar, 5 μm.

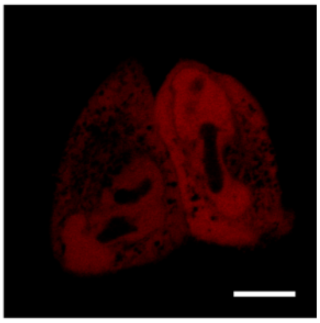

pmCherry C1 vector

Figure S6. Representative confocal image of HeLa cells transfected with pmcherry C1 empty vector. Scale bar, 5µm.
